# TIDB: a comprehensive database of trained immunity

**DOI:** 10.1093/database/baab041

**Published:** 2021-07-09

**Authors:** Yang Cao, Qingyang Dong, Dan Wang, Ying Liu, Pengcheng Zhang, Xiaobo Yu, Chao Niu

**Affiliations:** Department of Environmental Medicine, Tianjin Institute of Environmental and Operational Medicine, No.1 Dali Road, Heping District, Tianjin 300050, China; Department of Environmental Medicine, Tianjin Institute of Environmental and Operational Medicine, No.1 Dali Road, Heping District, Tianjin 300050, China; State Key Laboratory of Proteomics, Beijing Proteome Research Center, National Center for Protein Sciences (Beijing), Beijing Institute of Lifeomics, No.38 Life Science Park Road, Changping District, Beijing 102206, China; Department of Environmental Medicine, Tianjin Institute of Environmental and Operational Medicine, No.1 Dali Road, Heping District, Tianjin 300050, China; Department of Environmental Medicine, Tianjin Institute of Environmental and Operational Medicine, No.1 Dali Road, Heping District, Tianjin 300050, China; State Key Laboratory of Proteomics, Beijing Proteome Research Center, National Center for Protein Sciences (Beijing), Beijing Institute of Lifeomics, No.38 Life Science Park Road, Changping District, Beijing 102206, China; Department of Environmental Medicine, Tianjin Institute of Environmental and Operational Medicine, No.1 Dali Road, Heping District, Tianjin 300050, China

## Abstract

Trained immunity is a newly emerging concept that defines the ability of the innate immune system to form immune memory and provide long-lasting protection against previously encountered antigens. Accumulating evidence reveals that trained immunity not only has broad benefits to host defense but is also harmful to the host in chronic inflammatory diseases. However, all trained immunity-related information is scattered in the literature and thus is difficult to access. Here, we describe Trained Immunity DataBase (TIDB), a comprehensive database that provides well-studied trained immunity-related genes from human, rat and mouse as well as the related literature evidence. Moreover, TIDB also provides three modules to analyze the function of the trained-immunity-related genes of interest, including Reactome pathway over-representation analysis, Gene Ontology enrichment analysis and protein–protein interaction subnetwork reconstruction. We believe TIDB will help developing valuable strategies for vaccine design and immune-mediated disease therapy.

**Database URL:** http://www.ieom-tm.com/tidb

## Introduction

Traditionally, it has been known that immunological memory is an exclusive hallmark of the adaptive immune response. However, accumulated evidence shows that innate immune responses can also build long-lasting memory to protect against previously encountered antigens, which has been termed as ‘trained immunity’ by Netea (1–4). Over the past few years, many studies have proven that prototypical innate immune cells such as natural killer cells and monocytes/macrophages also display a long-term adaptation and enhanced responsiveness to certain stimuli after infection or vaccination (3–5). Trained immunity has changed our perspective of host defense and immunological memory and could provide a new strategy for vaccine design and disease therapy (6–9).

Studies show that trained immunity in different microenvironments can be either beneficial or harmful to the host. For example, BCG vaccination protects the host against heterologous infections and malignancies through trained macrophage, which is independent of adaptive immunity (10). However, cancer cells can also induce epigenetic reprograming in innate immune cells through many mediators (11). These trained cell-secreted cytokines such as IL-6 and TNF could increase tumorigenicity and the spread of metastases in oral squamous cell carcinoma and lung, kidney and breast cancer (12, 13).

One of the most important mechanisms in trained immunity is metabolic and epigenetic rewiring, which convert oxidative phosphorylation to aerobic glycolysis and result in a pro-inflammatory response (14). *Candida albicans*, β-glucan and BCG vaccination can induce epigenetic remodeling through different pathways (15–17), which include the dectin-1-dependent and NOD2-dependent pathway (18, 19). As described in previous reports, induction or suppression of trained immunity achieved through the modulation of these pathways could be therapeutically exploited in some diseases (19, 20). The mechanisms underlying the above phenomena related to trained immunity rely deeply on the variation of gene expression.

However, all trained immunity-related information is dispersed in the literature. A comprehensive set of well-annotated trained immunity-related genes would contribute significantly to the understanding of the role of trained immunity in health and disease. To meet this need, we built TIDB, which collects all previously identified genes associated with trained immunity from three species (human, rat and mouse) using text-mining and manual curation.

### Data collection and manual curation

**Figure 1.**
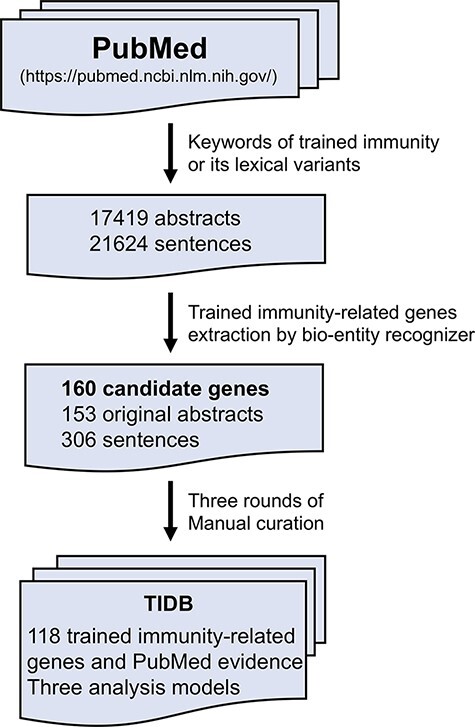
The workflow of TIDB construction.

TIDB is a manually curated database of trained immunity-related genes (Figure 1). Each entry is extracted from the PubMed abstracts using a text-mining method referred in Wang *et al.* (21). Briefly, the trained immunity-related literature was collected by searching in the field ‘title/abstract’ in PubMed database using keyword ‘trained immunity’ and its lexical variants, including ‘trained innate immunity’, ‘trained effect’, ‘training effect’, ‘trained host defense’, ‘immune innate memory’, ‘innate immune memory’, ‘immune training’, ‘immune trained’ and ‘immune non-specific memory’. The obtained literature abstracts were retrieved using National Center for Biotechnology Information (NCBI) E-Utilities Application Programming Interface (API) (22). Then, trained immunity-related genes were identified and extracted using a custom bio-entity recognizer based on protein ontology, and only genes from three species (human, rat and mouse) were kept in our database. Finally, all remaining genes and the evidence sentences in which the gene and keyword co-occurred were manually validated by three rounds of manual curation: (i) all sentences with trained immunity-related genes were checked and selected by two experienced researchers independently; (ii) all the selected sentences were manually reviewed and approved by three experts and (iii) all coauthors were asked to randomly check the data from the website to ensure the correctness and quality of our database. In addition, basic information (such as symbol, synonyms and location in genomes) of genes in TIDB was retrieved from the NCBI gene database, and evidence was annotated with terms from the Evidence & Conclusion Ontology (ECO) (23).

### Database overview and implementation

An overview of TIDB is shown in Supplementary Figure S1. TIDB has been designed as a user-friendly web interface where users can search, browse, download and analyze trained immunity-related genes and literature evidence of human, rat and mouse. The website interface comprises five main pages including ‘Home’, ‘Browse & Download’, ‘FAQ’, ‘Feedback’ and ‘Log in’. On the ‘Home page’, users can search for trained immunity-related genes. Then, users can perform three kinds of analysis, including Reactome pathway (24) over-representation analysis (ORA), Gene Ontology (GO) (25) enrichment analysis and protein–protein interaction (PPI) subnetwork reconstruction on genes of their interests. Users can browse and download all the trained immunity-related genes and the supporting literature evidence using the ‘Browse and Download’ page. The ‘FAQ’ page includes general questions and answers, which guide the users on how to use TIDB conveniently. On the ‘Feedback’ page, users can submit new genes to our database manually. Moreover, our website also provides manual annotation at the end of each evidence phrase, which requires user registration and login on the ‘Log in’ page.

TIDB is hosted on an Apache webserver with MongoDB, NodeJS and eggJS in the back-end. The dynamic, interactive front-end of our database is developed using HTML5, CSS3 and JavaScript. Bootstrap and jQuery framework are used to generate a responsive interface. Cytoscape.js is used for PPI network visualization. We recommend using the latest versions of Chrome, Firefox or Safari web browser for the best experience. The source code of TIDB is freely available under Creative Commons license (CC BY-SA 4.0) at https://github.com/niuclab/TIDB for possible contribution, discussion and change tracking.

### Data search and navigation

**Figure 2.**
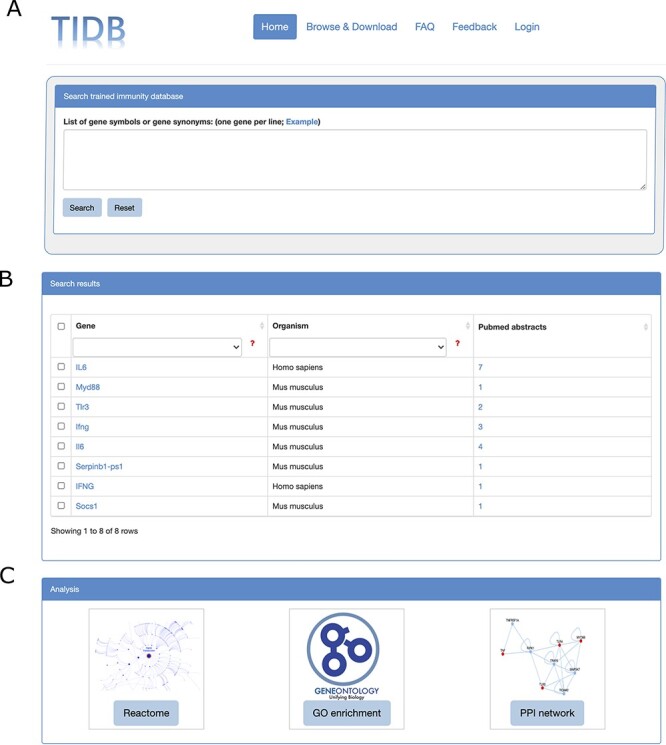
Screenshots of search box at home page (A), search results (B) and the three analysis modules (C).

**Figure 3.**
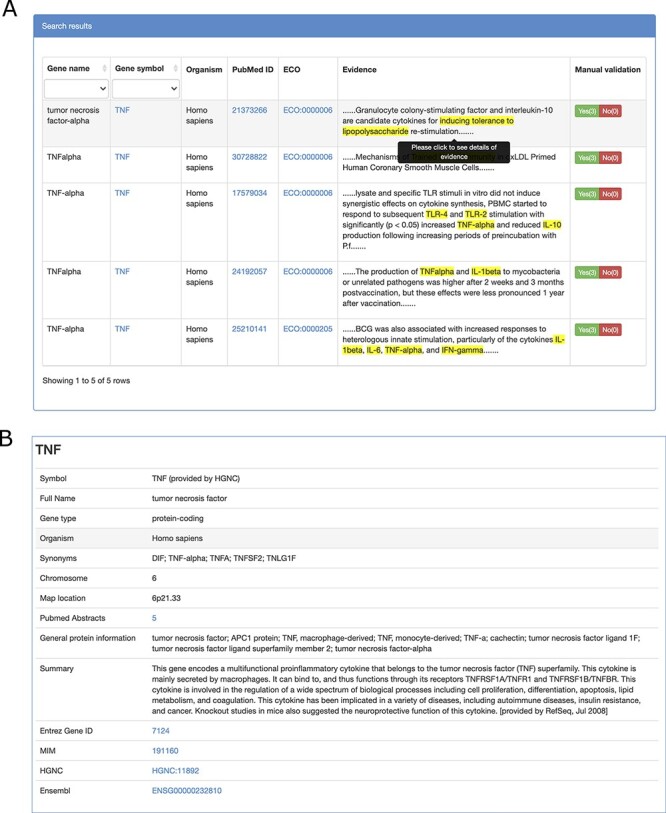
Screenshots of literature evidence page (A) and gene information page (B).

TIDB provides a user-friendly web interface that allows searching and exploring information on trained immunity-related genes (Figure 2A and B). Gene symbols or gene synonyms are supported by a query in TIDB. After clicking the ‘Search’ button, the search engine will run and return the queried results as a three-column table containing gene symbol, organism, and the supporting literature evidence. By clicking the hyperlink of a gene symbol in the ‘Gene’ column of the search results, the user can obtain basic information for the trained immunity-related gene and cross-references to external databases (i.e. NCBI Entrez (26) and Ensembl (27), Figure. 3B). When clicking the hyperlink of literature evidence, the whole abstract will be represented in which the evidence sentence, keywords and gene names are highlighted (Figure 3A). All information for trained immunity-related genes and their supporting literature evidence are listed in the ‘Browser & Download’ page and can be downloaded easily.

### Analyzer

**Figure 4.**
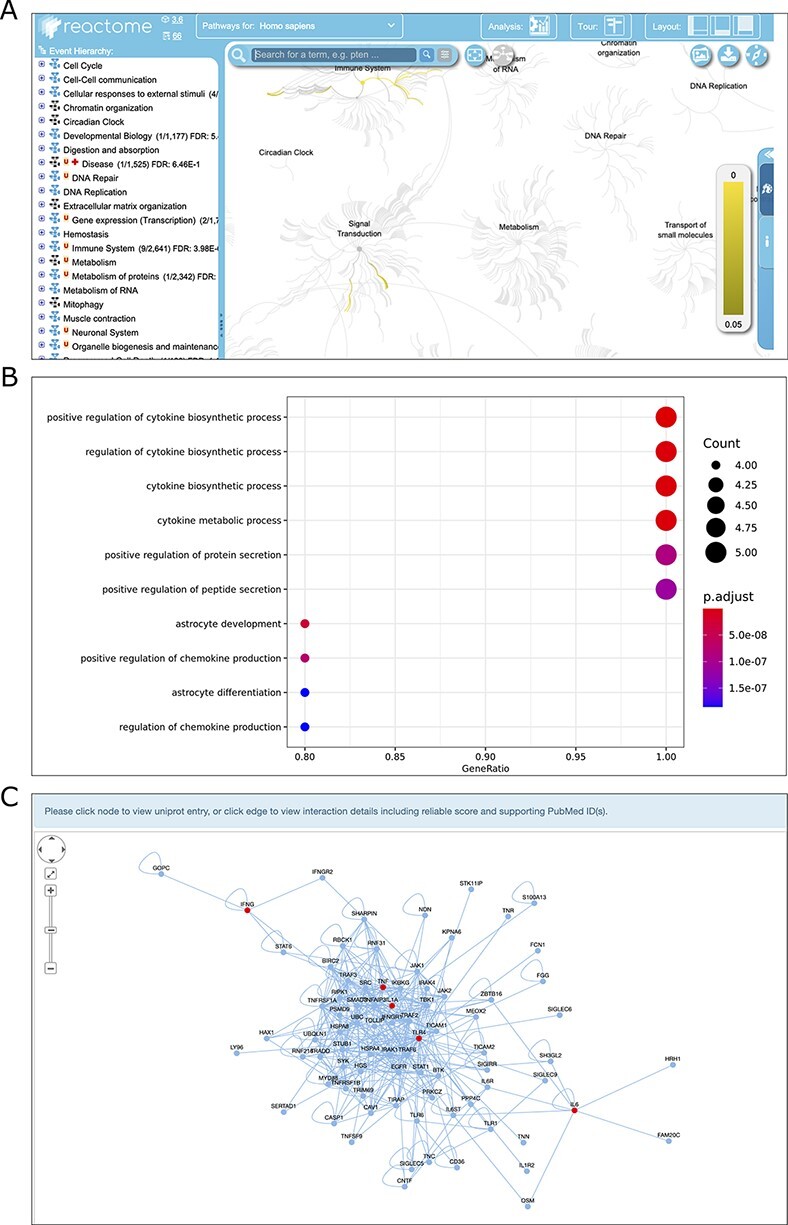
Screenshots of the results of three analysis modules, Reactome pathway ORA (A), GO enrichment analysis (B) and PPI network reconstruction (C).

Once the data search process is completed, by selecting the genes of interest from the search results, users can use three analytical tools to conduct further analysis and reveal the interesting results behind these genes (Figure 2C, Figure 4). Reactome pathway ORA and GO enrichment analysis were provided to test the probability that genes of interest are over-represented in Reactome pathways and GO terms PPI subnetwork reconstruction can be used to identify molecular interactions, predict protein functions, analyze functional modules and discover hub regulatory genes of disease (28). For more details on how to analyze the genes of interest and interpret the analysis results, see the manual of TIDB (http://www.ieom-tm.com/tidb/help).

### Reactome pathway ORA and GO enrichment analysis

Reactome pathway ORA and GO enrichment analysis were used to discover which pathways and GO terms are associated with the selected genes. The hypergeometric test (or Fisher’s exact test) followed by a correction for multiple hypothesis testing using the false discovery rate (FDR) method was employed to calculate the probability of enriched term.

Pathway ORA has a broad range of applications in life sciences and medical research. Reactome is an open source of manually curated pathway database for unveiling the mechanisms and small molecules involved in high-order biological pathways. TIDB integrates the pathway over-representation analysis via the Reactome web service. Once this analysis is complete, analysis results are shown in the ‘Reactome Analysis’ tab, within the ‘Details Panel’ (Figure 4A). All Reactome pathways are shown in blocks of 20 pathways, ranked by the *P*-value obtained from ORA.

GO is the most comprehensive and widely used knowledgebase for functional studies of genes. GO covers three distinct aspects of gene function, namely, biological processes (BP), cellular components (CC) and molecular functions (MF) (29). GO enrichment analysis has become a common method for the functional study of gene sets and can be used to gain insights into the BP they are involved in. This tool requires users to specify the species, the category of ontology (BP, CC or MF), P-value cutoff, and to check whether to perform the FDR correction for multiple hypothesis testing. The enriched GO terms will be visualized and a table containing the details of the enriched GO term, and IDs of genes of interest mapped to the GO term, *P*-value and q value (FDR corrected *P* value) will be displayed on the result page (Figure 4B). The details of the enriched GO terms can be downloaded as a csv-formatted file.

We examined the function of the trained immunity-related genes of human by performing Reactome pathway ORA and GO enrichment analysis. As a result, the most enriched Reactome pathways are cytokine interleukin-related pathways (e.g. interleukin-10 signaling, interleukin-4 and interleukin-13 signaling) and toll-like receptor (TLR) cascades-related pathways (e.g. TLR6:TLR2 Cascade, TLR 2 Cascade) (Supplementary Table S1); the most enriched GO BP terms are positive regulation of cytokine production, bacteria-related terms, (e.g. response to molecule of bacterial origin, response to lipopolysaccharide) and cellular response to biotic stimulus (Supplementary Figure S2). It should be mentioned that the cytokines are mainly secreted by innate immune cells (e.g. monocytes and macrophages), and TLR forms the cornerstone of the innate immune response (30). Moreover, receptors of bacteria, such as TLR and lipopolysaccharide, are mainly expressed on innate immune cells (31). These results suggest that the most enriched functional terms for trained immunity-related genes are all associated with innate immune response, which is consistent with above mentioned that trained immunity is a special program of innate immunity memory.

### PPI subnetwork reconstruction

PPI subnetwork analysis was implemented to uncover the insightful landscape of the molecular interactions and topological attributes for the selected trained immunity-related genes. For PPI subnetwork reconstruction, we first downloaded the reference PPI data of human, rat and mouse from the IntAct database (32). Then, the PPI subnetwork, which was centered on the seed proteins with the largest average clustering coefficient was constructed via random walking with restart (RWR) approach (33). This tool requires users to specify the number of random walk steps of RWR, the species and the edge weight cutoff used to subset the IntAct PPI network. As a result, the PPI network, in which the interest genes are filled in red, will display on the result page (Figure 4C). Moreover, the network can be saved as the SVG format, and the edge lists can be exported in CSV format.

### Feedback

TIDB provides a feedback feature that allows users to submit new trained immunity-related genes and the corresponding literature evidence manually on the ‘Feedback’ page. In addition, TIDB also provides manual annotation at the end of each evidence phrase, by which users can confirm the evidence or deny it by simply clicking the ‘Yes’ or ‘No’ button to confirm or reject the evidence phrase after login as registered users.

## Discussion and future directions

Increasing evidence has proven that trained immunity-related genes play an important role in health and disease, but the information of the previously identified trained immunity-related genes is scattered in numerous pieces of literature. In this study, we developed TIDB, an open source and comprehensive database of trained immunity-related genes. The trained immunity-related genes were identified using an in-house ontology-based bio-entity recognizer from the abstracts of all publications from PubMed with the keyword ‘trained immunity’ and its lexical variants. TIDB is the first extensive database that provides details on trained immunity-related genes in human, rat and mouse as well as the literature evidence and integrates three analysis modules (Reactome pathway ORA, GO enrichment analysis and PPI subnetwork reconstruction). It allows users to search and download information about trained immunity-related genes freely and easily. We believe that this database can contribute significantly to the understanding of the role of trained immunity in health and disease.

In the future, we will continue to identify and collect more trained immunity-related genes and improve TIDB to provide more accurate information. We will also update our database periodically to include feedback from the users.

## Supplementary Material

baab041_Supp
