# Supplementary material for: TIDB: a comprehensive database of trained immunity
Source: Database (Oxford). 2021 Jul 9;2021:baab041. doi: 10.1093/database/baab041 (PMC8271126; doi:10.1093/database/baab041)
Supplement: baab041_Supp [file baab041_supp.zip › supplementary_materials.docx]

**TIDB: a comprehensive database of trained immunity**

Yang Cao^1,#^, Qingyang Dong^1,#^, Dan Wang^2,#^, Ying Liu^1^, Pengcheng Zhang^1^, Xiaobo Yu^2,*^, Chao Niu^1,*^

1Department of Environmental medicine, Tianjin Institute of Environmental and Operational Medicine, Tianjin 300050, China, 2 State Key Laboratory of Proteomics, Beijing Proteome Research Center, National Center for Protein Sciences (Beijing), Beijing Institute of Lifeomics, Beijing 102206, China

^#^These authors contributed equally to this work.

^*^To whom correspondence should be addressed.


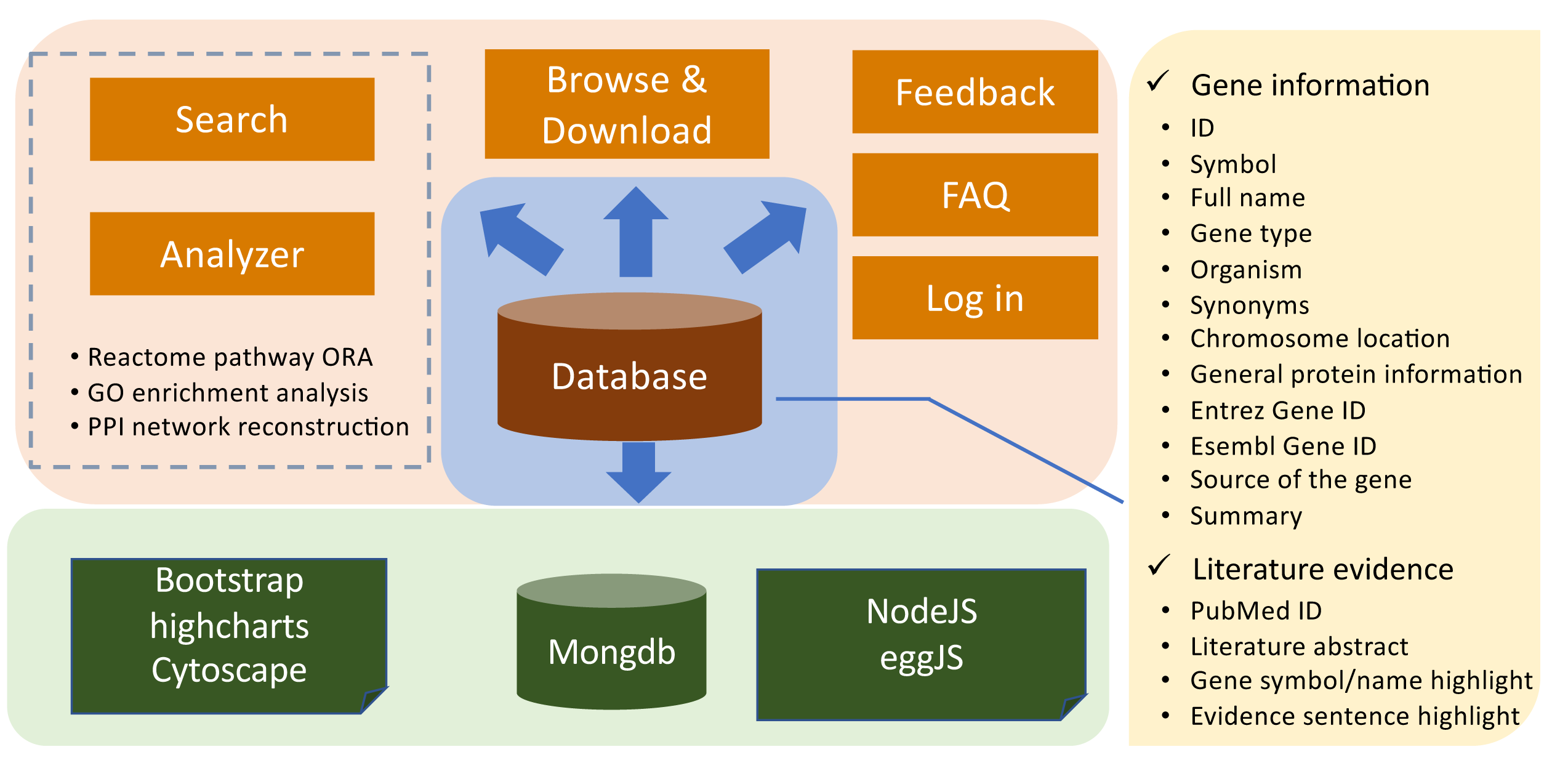


Supplementary Figure S1. Overview of TIDB.


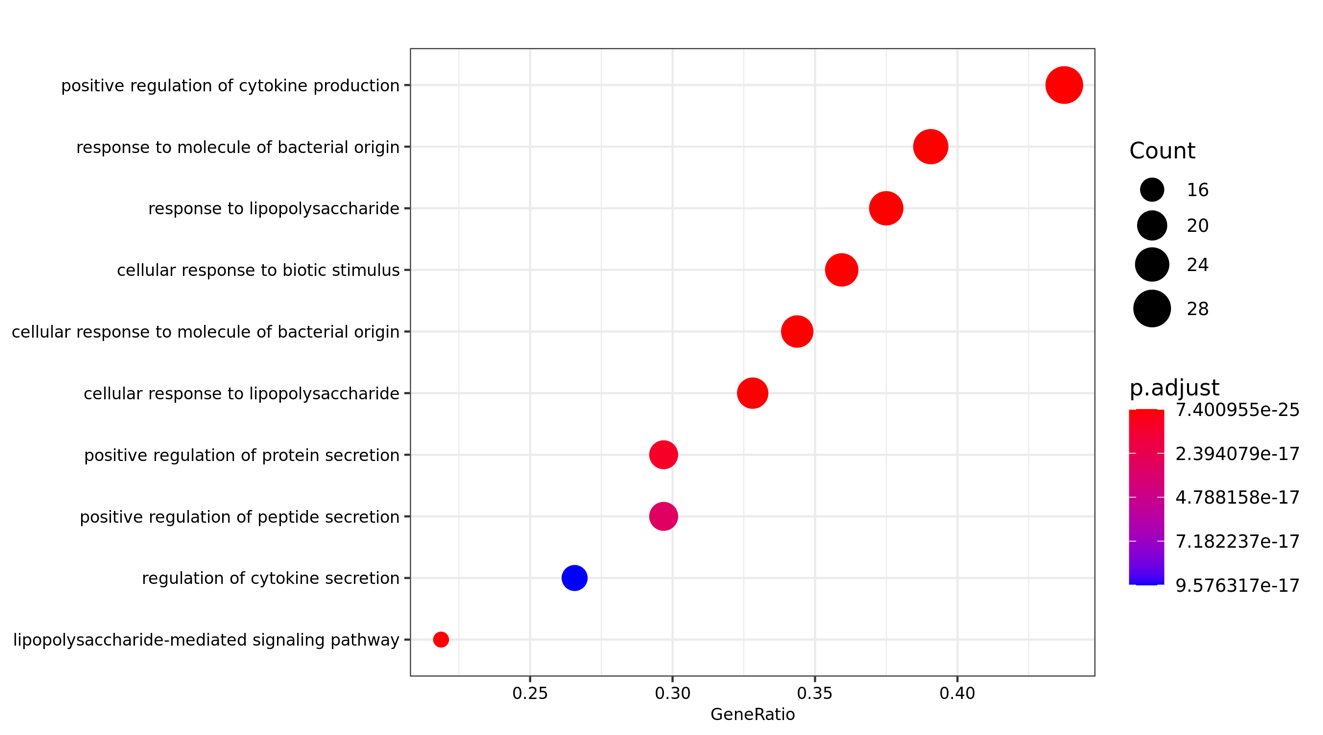


Supplementary Figure S2. Top 10 enriched GO terms of trained immunity-related genes of human.

Supplementary Table S1. Top 10 enriched Reactome pathways of trained immunity-related genes of human.

| **Pathway name** | **Entities found** | **Entities total** | **Entities ratio** | **Entities pValue** | **Entities FDR** | **Reactions found** | **Reactions total** | **Reactions ratio** |
| --- | --- | --- | --- | --- | --- | --- | --- | --- |
| Interleukin-10 signaling | 21 | 86 | 0.00615033 | 1.11E-16 | 1.32E-14 | 12 | 15 | 0.00127248 |
| Interleukin-4 and Interleukin-13 signaling | 26 | 211 | 0.01508975 | 1.11E-16 | 1.32E-14 | 22 | 46 | 0.00390227 |
| Signaling by Interleukins | 50 | 640 | 0.04576986 | 1.11E-16 | 1.32E-14 | 203 | 491 | 0.04165253 |
| Cytokine Signaling in Immune system | 53 | 1055 | 0.07544876 | 1.11E-16 | 1.32E-14 | 239 | 639 | 0.05420767 |
| Immune System | 68 | 2641 | 0.1888722 | 1.11E-16 | 1.32E-14 | 410 | 1493 | 0.12665422 |
| Toll Like Receptor TLR6:TLR2 Cascade | 12 | 110 | 0.0078667 | 3.11E-11 | 2.05E-09 | 45 | 65 | 0.00551408 |
| MyD88:MAL(TIRAP) cascade initiated on plasma membrane | 12 | 110 | 0.0078667 | 3.11E-11 | 2.05E-09 | 43 | 63 | 0.00534442 |
| Toll Like Receptor 2 (TLR2) Cascade | 12 | 113 | 0.00808124 | 4.22E-11 | 2.28E-09 | 47 | 67 | 0.00568375 |
| Toll Like Receptor TLR1:TLR2 Cascade | 12 | 113 | 0.00808124 | 4.22E-11 | 2.28E-09 | 45 | 65 | 0.00551408 |
| Toll Like Receptor 4 (TLR4) Cascade | 12 | 144 | 0.01029822 | 6.46E-10 | 3.17E-08 | 70 | 94 | 0.00797421 |
